# Cobalt-doped ZnO nanoparticles and PLD-deposited thin film forms: structure, optical properties and nature of magnetic anisotropy

**DOI:** 10.1039/d4ra05021e

**Published:** 2024-08-30

**Authors:** N. Khlifi, N. Ihzaz, O. Toulemonde, A. Dandre, C. Labrugère-Sarroste, M. N. Bessadok, O. M. Lemine, L. El Mir

**Affiliations:** a Laboratory of Physics of Materials and Nanomaterials Applied at Environment (LaPhyMNE), Gabes University, Faculty of Sciences in Gabes 6072 Gabes Tunisia nejib.ihzaz@issatgb.rnu.tn; b ICMCB-CNRS – Université de Bordeaux 87, avenue du Dr Albert Schweitzer 33608 Pessac cedex France; c PLACAMAT UMS 3625, CNRS, University of Bordeaux 33600 Pessac France; d Department of Physics, College of Sciences, Imam Mohammad Ibn Saud Islamic University (IMISU) Riyadh 11623 Saudi Arabia

## Abstract

Cobalt-doped zinc oxide nanoparticles (NPs) were synthesized using a modified sol–gel method. Thereafter, the obtained powder was deposited on a Suprasil glass substrate by employing a pulsed laser deposition (PLD) technique. X-ray diffraction analysis with Rietveld refinement confirmed a hexagonal wurtzite ZnO phase belonging to the *P*6_3_*mc* space group for both samples in the NP and thin film forms. In particular, the thin film exhibited an intensive (002) XRD peak, indicating that it had a preferred *c*-axis orientation owing to the self-texturing mechanism. No segregated secondary phases were detected. The crystallite structure, morphology, and size were investigated using high-resolution transmission electron microscopy (HRTEM). To study the crystalline quality, structural disorder, and defects in the host lattice, we employed Raman spectroscopy. UV-vis-NIR spectroscopy was performed to confirm the nature of the Co-doped ZnO NP powder and the film. The chemical states of oxygen and zinc in the thin film sample were also investigated *via* X-ray photoelectron spectroscopy (XPS). The *M*–*T* curve could be successfully fitted using both the three-dimensional (3D) spin-wave model and Curie–Weiss law, confirming the mixed state existence of weak ferromagnetic (FM) and paramagnetic (PM) phases. Magnetic interaction was quantitatively studied and explained by polaronic percolation of bound magnetic polarons (BMPs). Analysis of magnetic symmetry of the topological antiferromagnetic as-deposited thin film using torque measurements was performed. Based on a phenomenological model, it was revealed that the structure gives rise to uniaxial magneto-crystalline anisotropy (UMA) with the magnetic easy axis parallel to the *c*-axis.

## Abstract 2

Cobalt-doped zinc oxide nanoparticles (NPs) were synthesized using a modified sol–gel method.

## Graphical abstract

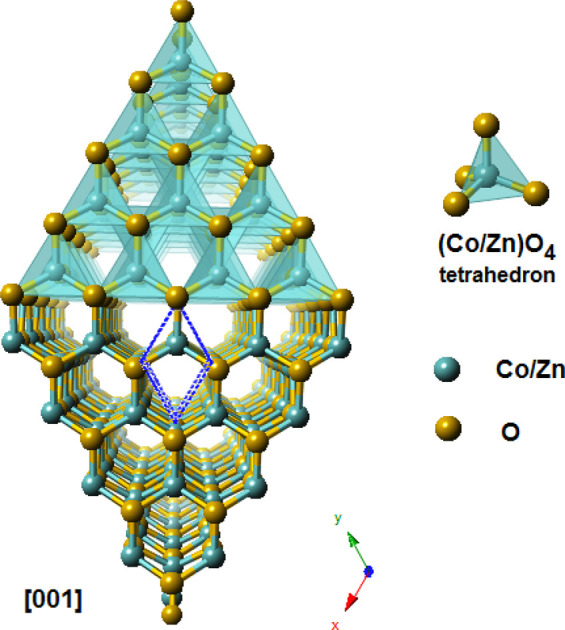

## 1. Introduction

Zinc oxide (ZnO) has attracted significant attention as a semiconductor owing to its multifaceted characteristics and has found applications in various technological fields, such as electronics, optoelectronics, and spintronics.^1–8^ This compound possesses a large energy band gap of 3.37 eV and a wide exciton binding energy of 60 meV. It is used in many applications, such as in light-emitting diodes, photodetectors, and gas sensors.^5^ The theoretical prediction of ferromagnetism at room temperature (RTFM) in ZnO doped with transition metals (TMs) and the discovery of ferromagnetism in pure ZnO and in TM-doped ZnO, such as ZnO doped with Mn, Ni, and Co, have presented an opportunity to utilize these diluted magnetic semiconductors (DMSs) in the field of magneto-optics and magneto-electronics (spintronics) technologies. It has been shown that TM-doped ZnO, synthesized using various techniques, displays diverse magnetic characteristics ranging from paramagnetism to ferromagnetism. The Curie temperature (*T*_c_) associated with these materials varies between 20 K and 550 K.^9^ Cobalt has attracted significant interest as a transition metal-doped ZnO. This interest stems not only from its room temperature ferromagnetism (RTFM)^10–12^ but also from the fact that Co exhibits a high solubility limit in ZnO and possesses a large magnetic moment per Co ion.^13–16^ It is worth noting that the cobalt ion Co^+2^ has a similar radius to that of the Zn^+2^ ion, resulting in a fortuitous matching of their ionic radii and creating a unique combination of materials. In this scenario, it is anticipated that Co-doped ZnO will possess a crystal structure identical to ZnO and is likely to form a single phase with Co^+2^, substituting the Zn^+2^ positions. Following the theoretical proposal by Sato *et al.*^17^ that RTFM (room temperature ferromagnetism) could be achieved in Co-doped ZnO, and the subsequent experimental discovery of RTFM in Co-doped ZnO thin films,^18,19^ a significant number of experimental findings concerning Co-doped ZnO have been published over the past decade.^20–24^ Despite several papers being published in this area, there is a lack of consensus among different research groups regarding the magnetic properties of Zn_*x*_Co_1−*x*_O nanocrystalline particles prepared using various methods.^25–27^ The question of whether the observed RTFM behavior in Zn_*x*_Co_1−*x*_O is a result of intrinsic effects (mediated by super-exchange at the atomic scale and charge carriers at a larger scale), or if it is only induced by secondary phases or interfaces remains unanswered. Furthermore, a complete understanding of the relationship between the magnetic and optical behavior in these nanocrystalline particles is also lacking, as contradictory results have been reported in the literature.^28^

In this contribution, we provide detailed information about the characteristics of 5% Co-doped ZnO, which was synthesized in the form of nanoparticles using a modified sol–gel method, as well as in the form of thin films deposited on a Suprasil substrate using a pulsed laser deposition (PLD) technique. We aimed to gain a deeper understanding of the factors responsible for the emergence of the ferromagnetic (FM) component in these nanocrystalline particles and to explore the relationship between their magnetic and optical properties.

## 2. Experimental

In the initial step, we prepared Zn_1−*x*_Co_*x*_O (*x* = 0.05) NPs (hereafter called ZCO) by a modified sol–gel method. Here, 16 g of zinc acetate dihydrate [Zn (CH_3_COO)_2_·2H_2_O] was dissolved in 112 mL methanol. After 10 min magnetic stirring at room temperature, an adequate quantity of cobalt acetate [(CH_3_COO_2_)CO·4H_2_O] was added. After 15 min magnetic stirring, the solution was placed in an autoclave. Then, it was dried in a supercritical condition of ethyl alcohol (EtOH) (*T*_c_ = 250 °C, *P*_c_ = 80 bar) following the protocol described by El Mir *et al.*^29,30^ after adding 188 mL of EtOH. The produced nanopowder was calcined in an open oven for 2 h at 400 °C. In the second step, Co-doped zinc oxide thin films were deposited on suprasil substrates by PLD (PLD/MBE 2100) from PVD products obtained using a KrF excimer laser (*λ* = 248 nm, pulse width 20 ns, and repetition rate = 10 Hz) operated at 350 mJ to ablate the target. The deposition temperature and pressure were 300 °C and 10^−6^ torr, respectively. The substrate–target distance was 55 mm. All the PLD parameters that could influence the deposition rate were kept constant during the deposition period.^31^ The structural properties of the synthesized powder and thin film were investigated by X-ray diffraction (Bruker AXS B8 Advance using Cu Kα radiation). The chemical composition was investigated by X-ray photoelectron spectrometry (XPS) using a Thermo-Fisher Scientific K-Alpha spectrometer equipped with a mono-chromatic Al Kα source (*hν* = 1486.6 eV) and a 400 μm X-ray spot size. The morphology of the samples was studied with a JEM-200CX transmission electron microscopy (TEM) system. The Raman spectra were obtained at room temperature using a Microscope confocal Raman Thermo-Fisher DXR system (3 lasers: 532, 633, 785 nm). The optical properties were investigated by UV-visible-IR spectrometry (Shimadzu UV-3101PC) coupled with an integrated sphere in the wavelength range from 200 to 2400 nm. Magnetic measurements were performed by VSM on a Microsense EZ-7 and SQUID Quantum Design MPMS-7X system. The thickness of the films was evaluated using a Tencor profilometer. The surface morphology and roughness were characterized by atomic force microscopy (AFM, TopoMetrix) and scanning electron microscopy (SEM, HITACHI S4500).

## 3. Results and discussion

### 3.1 XRD and Rietveld analysis

**Fig. 1:**
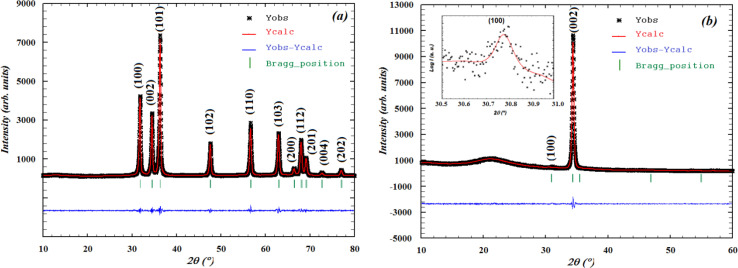
Rietveld analysis plots realized with FULLPROF software for X-ray diffraction patterns at room temperature for the (a) ZCO NPs and (b) ZCO thin film.

**Fig. 2:**
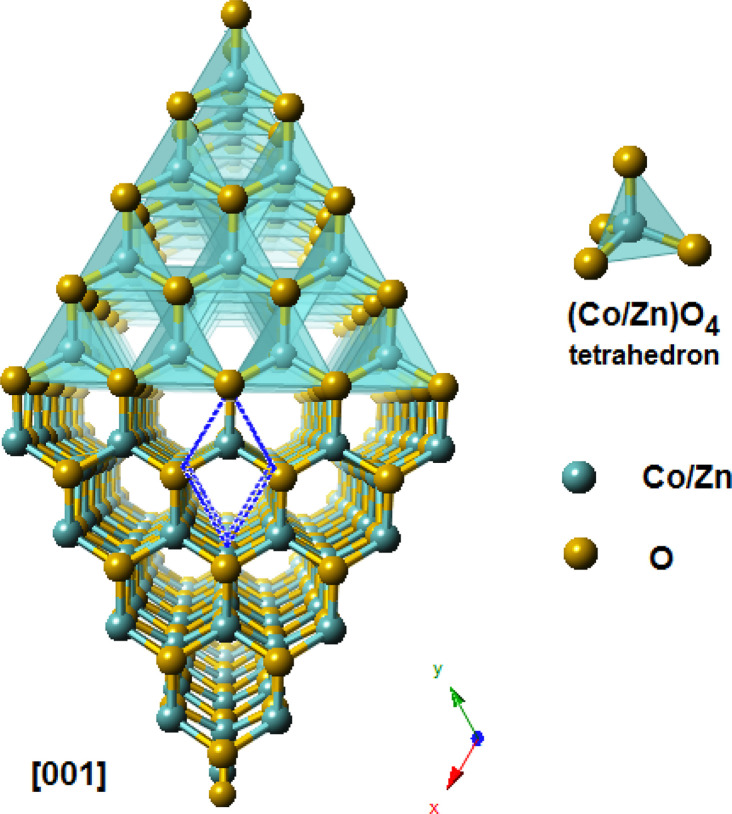
A view in perspective of the 3D and polyatomic ZnO nanoparticles for better understanding the crystal structure with a preferential orientation in the (002) direction, indicating that the *c*-axis was perpendicular to the film surface.

The Rietveld diagrams for the XRD data of the ZCO NPs powder and the thin film acquired at room temperature and inputted into the FULLPROF software^32^ are displayed in Fig. 1. It is evident that the as-prepared nanopowder was well crystallized, and could be finely indexed to a wurtzite structure with a pure hexagonal symmetry unit cell (*P*6_3_*mc* space group), presenting satisfactory convergence factors. A very precise fit among the observed and calculated powder patterns was provided by the Rietveld fit. The observed pattern is shown by the dots in Fig. 1(a), while the predicted pattern is shown by the solid line. The lower line shows the discrepancy between the calculated and observed XRD diffractograms. The vertical bars represent the Bragg peak positions. Fig. 1(b) shows the XRD pattern for the thin film. The diffractogram revealed the existence of a ZCO single phase with a hexagonal wurtzite structure, showing a preferential orientation in the (100) direction with a low-intensity peak, as shown in the inset in Fig. 1(b) and in the (002) direction with the major peak indicating that the *c*-axis was perpendicular to the film surface. Furthermore, the preferential orientation along the (002) plane that is evident in the ZCO grown on the suprasil substrate was assisted by the presence of non-bridging oxygen atoms in the substrate.^33^ The diffraction peak at (002) is a frequently observed phenomenon in ZnO that is obtained with solution-based methods,^34–38^ indicating that the (002) plane possessed the lowest surface free energy in the PLD-synthesized ZCO thin films. Consequently, this plane becomes the preferred orientation for the growth process. The diffraction pattern did not reveal the existence of any phase impurity during the film growth by the pulsed laser technique. The crystallographic refined structural parameters and reliability factors are provided in Table 1. They agree with the single-crystal X-ray diffraction data and the refined parameters in work previously reported by other groups.^36,37^ In Fig. 2, a view is provided in perspective of the 3D and polyatomic ZnO nanoparticles to aid a better understanding of the crystal structure with the preferential orientation in the (002) direction, indicating that the *c*-axis was perpendicular to the film surface. This explains the spatial arrangement of the (Co/Zn)O_4_ octahedra. The crystal structure for the deposited thin film was established using the “CrystalMaker” program^39^ based on the refined atomic positions found by XRD.

**Table 1:** Structural parameters of both the ZCO NPs and thin film sample at room temperature obtained from Rietveld refinement

Synopsis						
Formula			Zn_0·95_Ca_0·05_O			
Crystal system			Hexagonal wurtzite			
Space group			*P*6_3_*mc*			
			Nanopowder		Thin film	
Density (g cm^−3^)			5.940		5.607	

**Lattice parameters**						
*a* = *b* (Å)			3.25024 (5)		3.3436 (9)	
*c* (Å)			5.20465 (12)		5.2222 (17)	
*α* = *β* (°)			90			
*γ* (°)			120			
Volume, *V* (Å^3^)			47.616 (2)		50.563 (26)	

**Atomic coordinates**						
Atoms	Wyckoff	Sites	*x*	*y*	*z*	Occ.
Zn	2b	3m	0.33333	0.66667	0.50000	0.95
Co	2b	3m	0.33333	0.66667	0.50000	0.05
O	2b	3m	0.33333	0.66667	0.88005_Nanopowder_	1
					0.88227_Thin film_	

**Orientation factors**						
Nanopowder			0.004 (4)			
Thin film			0.217 (33)			

**Agreement factors**						
			*R*_p_ (%) = 4.27		*R*_p_ (%) = 3.38	
			*R*_wp_ (%) = 5.72		*R*_wp_ (%) = 4.32	
			*R*_exp_ (%) = 4.69		*R*_exp_ (%) = 4.66	
			*χ* ^2^ = 2.4		*χ* ^2^ = 1.68	

### 3.2 Morphological analyses

**Fig. 3:**
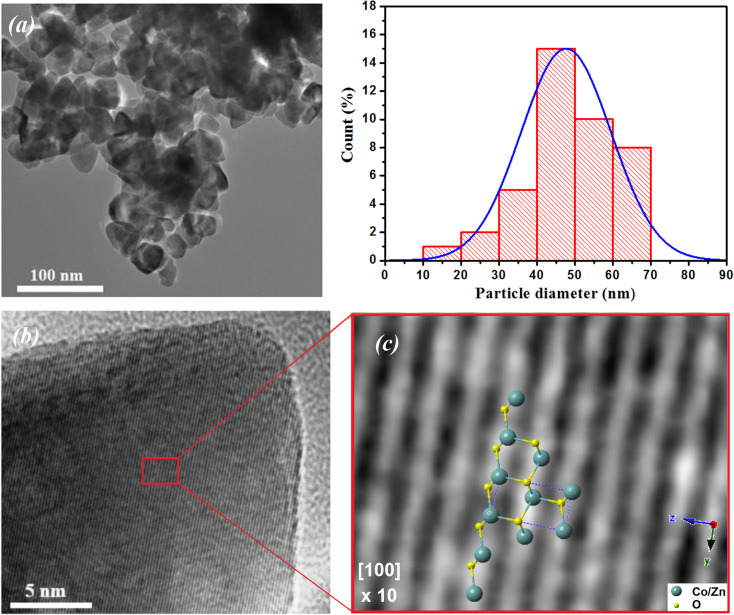
(a) TEM images of ZCO NPs along with their particle size distribution histogram. (b) HRTEM image and an enlarged region showing the structured Co-doped ZnO phase. (c) Schematic reconstruction of the unit cell and Co/ZnO_4_ tetrahedral distortions in the (100) axis.

**Fig. 4:**
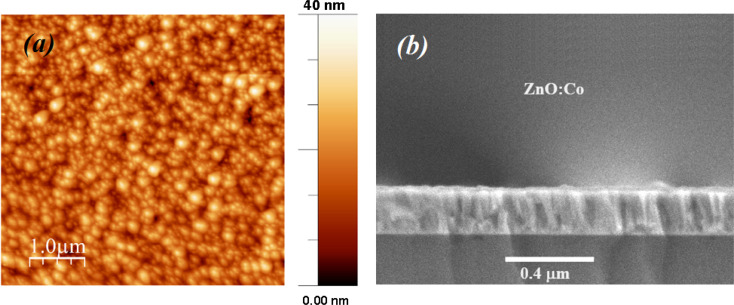
(a) AFM surface topology (b) and cross-section SEM images of the ZnO:Co thin film.

Fig. 3(a) shows the morphology and an estimation of the particle size according to the TEM image of the ZCO NPs. The particle size was estimated using ImageJ software *via* the histogram distribution function. It indicated irregular nanoparticles of various sizes with almost spherical shapes that were uniformly distributed. The majority of the particles revealed an average size of 47 nm. In addition, it was observed that these particles in the nanometric scale included grain boundaries and free surfaces, which may affect their physical properties.^40–42^Fig. 3(b) displays the high-resolution (HR) TEM image and an enlarged HRTEM image of the ZCO phase on a structured part of the particle region. The image of the contrast was compatible with the symmetry of the wurtzite ZnO phase nanostructure belonging to the *P*6_3_*mc* space group, simulated along the [100] direction. Herein, a schematic reconstruction of the unit cell and Co/ZnO_4_ tetrahedral distortions in the (100) axis is shown. The structural representation and the associated models were performed in the crystallographic simulation software CrystalMaker.^39^Fig. 4 depicts AFM and SEM cross-sectional images of the ZCO thin film, which demonstrated a preferential crystallographic orientation in the (002) plane, as proved by the highest peak intensity. It could be noticed that the film presented a uniform grain size distribution with a typical columnar structure, presenting a very smooth surface with a root mean square (RMS) roughness of about 12 nm. The thickness of the film was about 300 nm, with this value confirmed by Tencor profilometer measurements.

### 3.3 Raman spectroscopic behavior

**Fig. 5:**
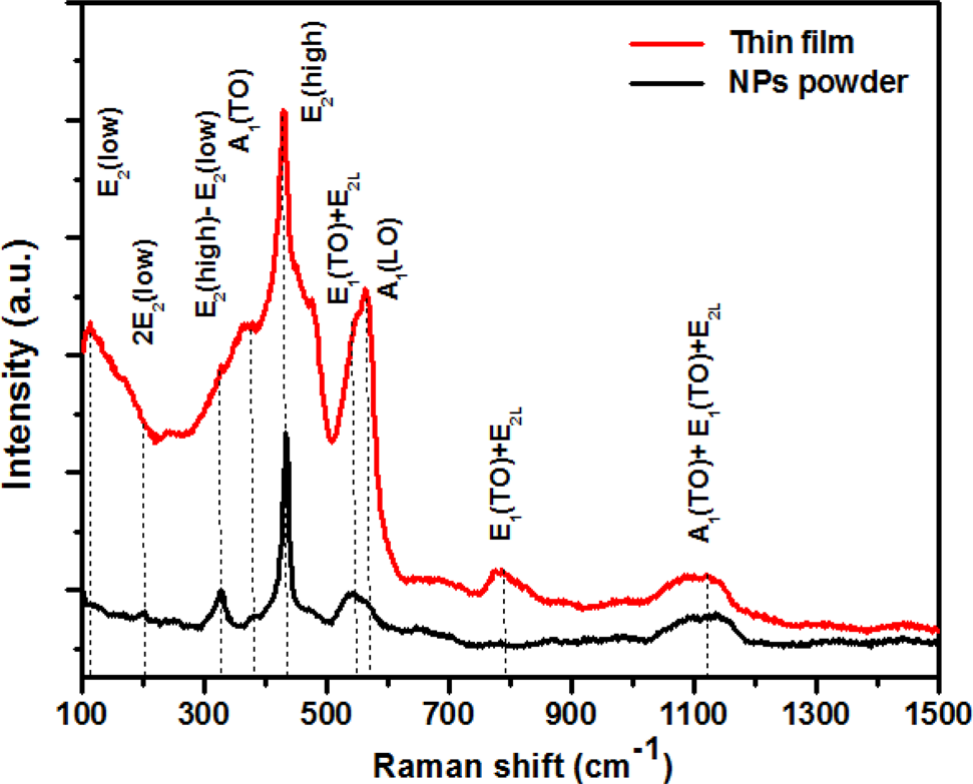
Room temperature Raman spectra of both ZCO NPs and the thin film sample.

Raman spectroscopy was employed for the examination and exploration of the growth process of the original crystal structure, the identification of oxygen vacancies, and the detection of localized defects within the nanoparticles. Fig. 5 shows the Raman spectra acquired by exciting the ZCO powder and thin film nanoparticles by a laser emitting at a wavelength of 532 nm. The examined sample possessed a wurtzite structure and was categorized under the space group *P*6_3_*mc*. The phonon mode was weakened on the ZCO powder with respect to the thin film, but the peak broadening was about the same, suggesting that there was not much deterioration of the lattice. As expected, the thin film was highly anisotropic, contrary to the powder, which showed a random crystal orientation to the direction of the incoming light. As per group theory, this specific crystallographic phase exhibited optical phonon modes at the center of the zone, which can be denoted as:^43^

1 *Γ*_Opt_ = A_1_ + 2B_1_ + E_1_ + 2E_2_

The aforementioned system was composed of a single A_1_ branch, two B_1_ branches, one E_1_ branch, and two E_2_ branches. It is noteworthy that among these branches, E_1_, E_2_, and A_1_ could be classified as first-order Raman active modes, as indicated by previous research.^44^ Each active vibration mode in the Raman spectra is depicted by a unique band, allowing for the identification and characterization of these modes. The intensity displayed by these bands can be determined by the scattering cross-section of the respective modes.^45^ The B_1_ modes do not demonstrate any infrared or Raman activity. Within this set of modes, both A_1_ and E_1_ possess polar symmetry and can be divided into transverse optical components known as A_1_(TO) and E_1_(TO), as well as longitudinal optical components called A_1_(LO) and E_1_(LO). The E_2_ mode is composed of two distinct modes: E_2_(low) and E_2_(high), which correspond to low- and high-frequency phonons, respectively; E_2_(high) is related with the oxygen atom, while E_2_(low) is linked to the Zn sub lattice. Both E_2_(low) and E_2_(high) modes exhibit Raman activity and are non-polar. Distinct peaks were observed in the ZCO NPs powder at specific wave numbers of 101, 200, 325, 383, 429, 543, 563, 782, and 1121 cm^−1^. Similarly, the ZCO thin film displayed notable peaks at 114, 200, 327, 382, 430, 545, 563, 783, and 1121 cm^−1^. Analysis of the results revealed that the ZCO thin film exhibited a shifted peak at 114 cm^−1^, suggesting a higher intensity compared to the powder sample. This shift could be attributed to the presence of structural defects, such as local lattice distortion, within the thin film.^46^ Additionally, the oxygen defect states were assessed based on the Raman peak intensity ratio, which was found to be approximately 563 cm^−1^. The intensity ratios for the ZCO NPs powder and the thin film were determined to be 0.40 and 0.69, respectively. These results indicate that the presence of oxygen defect states was more prominent in the thin film form than in the ZCO NPs powder.^47^

### 3.4 Optical analyses

**Fig. 6:**
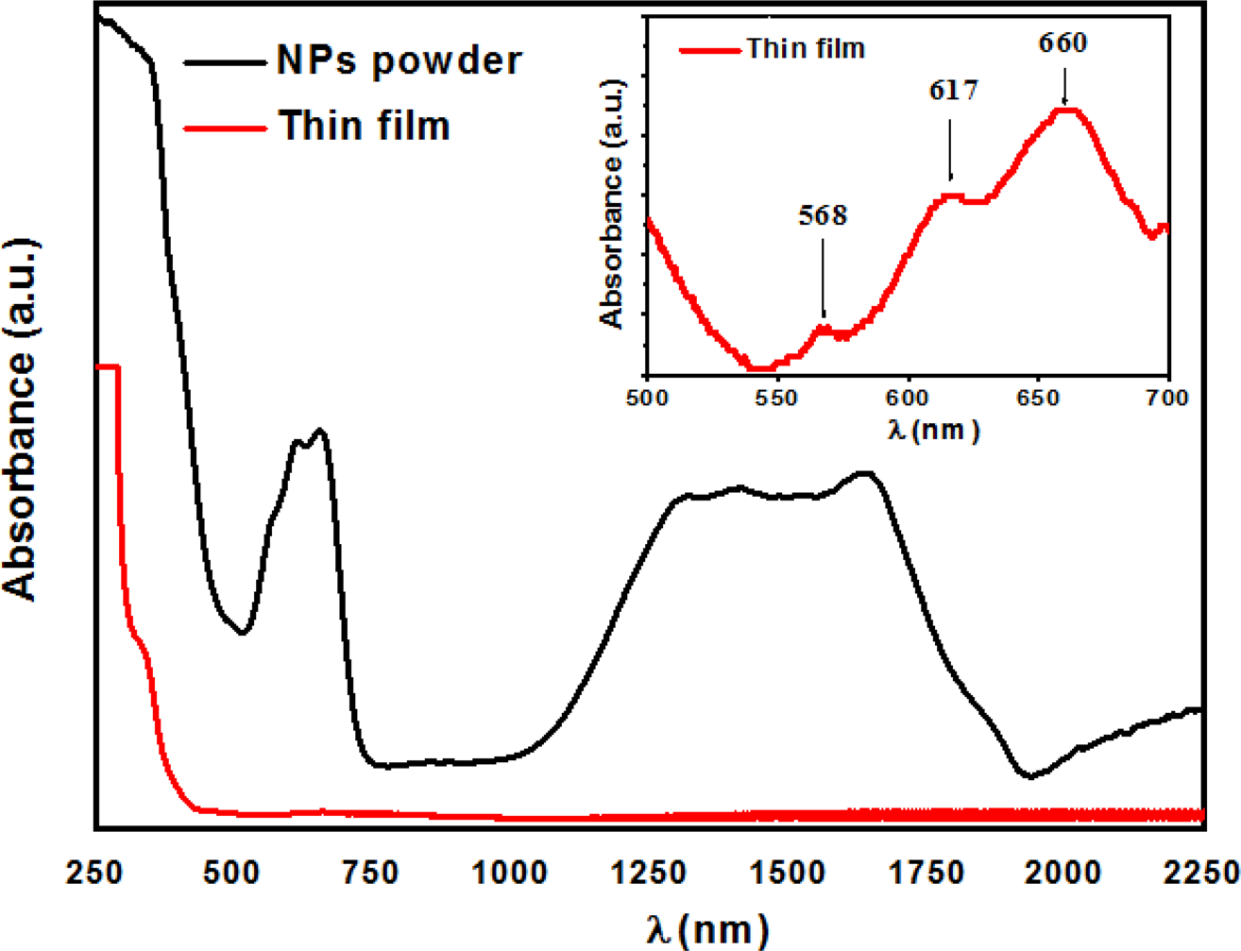
Absorption spectra of both the ZCO NPs and thin film sample.

**Fig. 7:**
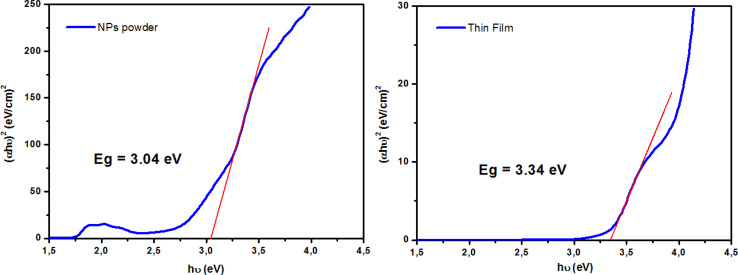
Tauc plots for determining the optical band gap (*E*_g_) for both ZCO NPs and thin film samples.

The optical properties of the ZCO NPs powder and thin film form were studied using UV-Vis-NIR absorption (250–2250 nm). As shown in Fig. 6, Co-related absorbance peaks were found between 550 and 700 nm in both the ZCO NPs powder and thin film sample. The pure ZnO samples displayed an absorption band edge at 393 nm, which suggested a direct bandgap of 3.16 eV.^48^ The introduction of Co ions in ZnO through the substitution of Zn^2+^ by Co^2+^ ions yielded three additional absorption bands centered at 568 nm (2.18 eV), 617 nm (2.01 eV), and 660 nm (1.87 eV), respectively. These peaks were related to the d–d (3d^7^) electronic transition of the tetrahedral-coordinated Co^2+^.^49^ The three absorption bands could be attributed to ^4^A_2_(F) → ^2^A_1_(G), ^4^A_2_(F) → ^4^T_1_(P), and ^4^A_2_(F) → ^2^E(G) ligand field transitions, respectively, which involved a crystal-field split in the 3d levels of Co^2+^ substituting for Zn^2+^ in ZnO.^50^ In the NIR region, the NPs powder sample displayed three peaks located at 1315 nm (0.94 eV), 1400 nm (0.88 eV), and 1645 nm (0.75 eV), which were assignable to ^4^A_2_(F) → ^4^T_1_(F). These absorption peaks in the vis-NIR were attributed to the d–d transition crystal field from the ^4^A_2_(F) state to the higher energy state of Co^2+^, as reported by Koidl *et al.*^51^ Due to the poor signal, these peaks were absent for the thin film. The optical gap for both samples was evaluated using the Tauc relation (eqn (2))^52^ by plotting (*αhν*)^2^*versus* (*hν*), as shown in Fig. 7 and by the equation below:2
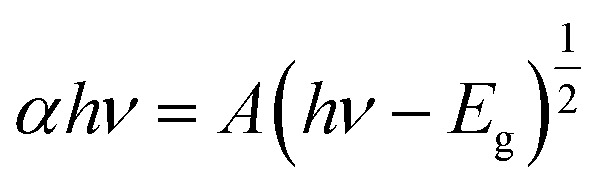
where *A* is a constant, *α* is the absorption coefficient, and *hν* is the photon energy. The extrapolation of the linear part of the curve to the abscissa axis provides the value of the band gap energy *E*_g_ of each sample. The expected *E*_g_ values were 3.04 and 3.34 eV for the NPs powder and thin film, respectively. For the NPs powder, the lower value compared to the pure ZnO sample could be explained by the sp–d exchange interactions between the band electrons in ZnO and the localized d electrons of Co^2+^.^53–55^ This behavior was not characteristic of the thin film sample due to the textured state of the ZnO matrix and therefore the absence of defects that are typically responsible for the exchange interactions.

### 3.5 XPS analysis

**Fig. 8:**
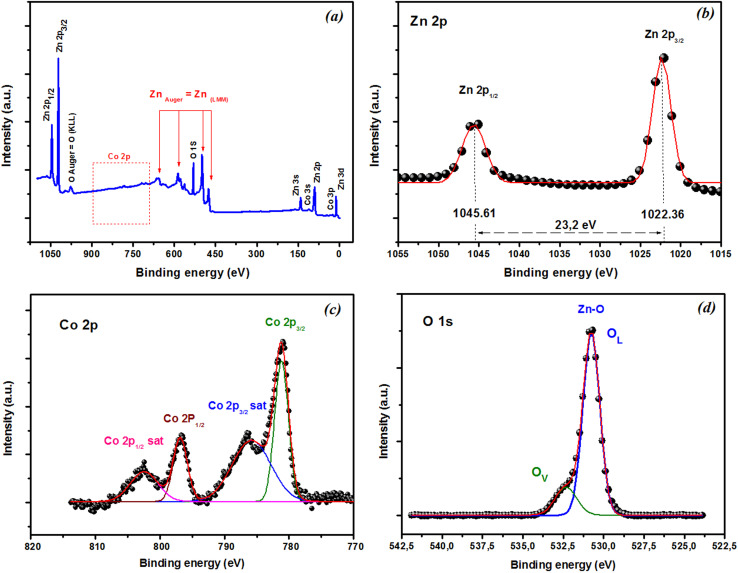
XPS spectra of CZO films. (a) XPS survey scan spectra. High-resolution XPS of (b) Zn 2p, (c) Co 2p, and (d) O 1s state energies.

XPS was used to explore the chemical states of the elements in the film. The XPS survey scan spectra in Fig. 8(a) showed that all the peaks could be assigned to Zn, O, and Co, and no impurities were observed in the film. Fig. 8(b) reveals that the Zn 2p peak was very sharp, with corresponding binding energies of Zn 2p_3/2_ and 2p_1/2_ around 1022.36 and 1045.61 eV, respectively. These findings suggest the presence of Zn in the oxidation state Zn^2+^. The difference in binding energy between these peaks was about 23.2 eV and was consistent with that of ZnO.^56^ XPS was also used to investigate the oxidation state of the Co ions in the films. The Co 2p spectra of the as-grown thin film deposited are shown in Fig. 8(c). The spectrum exhibited four Co 2p peaks: 2p_3/2_, 2p_1/2_ doublet, and their two satellite peaks at higher binding energies. The binding energy for Co 2p_3/2_ was 781.22 eV, and the energy difference between Co 2p_3/2_ and Co 2p_1/2_ was 15.6 eV, which matched with that of the standard CoO. These findings suggest the presence of Co in the as-grown thin films in the form of a Co^2+^ oxidation state.^57,58^ Generally, non-stoichiometric phases, including oxygen vacancies, are commonly present and widely observed in oxides,^59^ especially in oxide thin films produced through pulsed laser deposition (PLD). A subsequent annealing in oxygen was required to reduce the oxygen nonstoichiometry. To verify if the intrinsic nature of the exchange interaction in ZCO nanoparticles, which is studied in the following, was induced by oxygen defects, high-resolution XPS measurements were adopted to characterize the situation of oxygen, as displayed in Fig. 8(d). The high-resolution O 1s XPS spectra of the film could be fitted by two peaks: one located at 530.77 eV (marked by O_L_), attributed to the lattice O^2−^ anion in the Zn–O bond, and another at 532.30 eV (marked by O_V_), related to the oxygen defects.^60^ The relative concentration of oxygen near oxygen vacancies (O_V_/(O_V_ + O_L_)) can be roughly semi-quantitative by its area ratio in the O 1s XPS curves, which could be expressed as 17% of O_V_ and 83% of O_L_. The results suggest that the doping by Co^2+^ can enhance oxygen vacancies, which is beneficial for the formation of ferromagnetic coupling and for improving the magnetism in ZnO.

### 3.6 Magnetic characterization

**Fig. 9:**
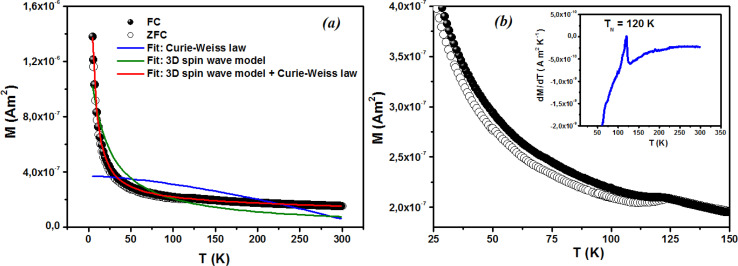
Magnetization of the ZCO thin films as a function of temperature in a field of 1000 Oe for (a) zero-field cooling (white circle symbols) and field cooling (black circle symbols). The fitting results of the field-cooled data are shown by the solid curves. (b) Enlarged parts for the temperature range of 25–150 K; the inset shows the relative difference d*M*/d*T vs. T* plot.

**Fig. 10:**
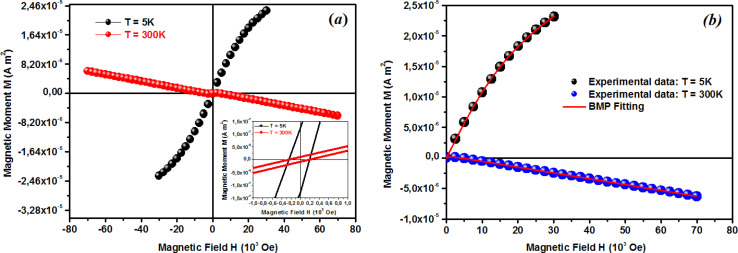
(a) Low and room temperature *M*–*H* plots showing the hysteresis loop for ZCO thin films. (b) Initial curve (0–*H*_ma*x*_) of the *M*–*H* plot fitted with the BMP model (eqn (6)) for the ZCO thin films. Symbols are for the experimental data and the solid line is the fit with the BMP model. Extracted parameters are shown in Table 2.

Fig. 9(a) displays the relationship between the magnetization (*M*) and temperature (*T*) for a thin film of ZCO under zero-field-cooled (ZFC) and field-cooled (FC) conditions. To determine the magnetization value under FC conditions (FCW mode), the sample was cooled from 300 K to 5 K with an applied field of 1000 Oe. Data were recorded during the subsequent temperature increase. To obtain the magnetization value under ZFC conditions, the sample was cooled from 300 K to 5 K without the presence of any magnetic field. Again, data were recorded during the temperature increase. Fig. 9(b) shows that the magnetization curves under the FC and ZFC conditions overlapped, but a closer examination reveals that the FC and ZFC magnetization values differed by approximately 120 K and showed a small increase, as depicted in the inset of Fig. 9(b). The *M*–*T* curve demonstrated that ZFC magnetization gradually increased as the temperature decreased from approximately 300 K to around 50 K. However, below 50 K and down to 5 K, the magnetization rapidly increased with further reductions in temperature. Our attempt to fit the *M*–*T* curve of the ZCO thin film involved the utilization of the Curie–Weiss (C–W) law,^61^ as given by the following equation:3
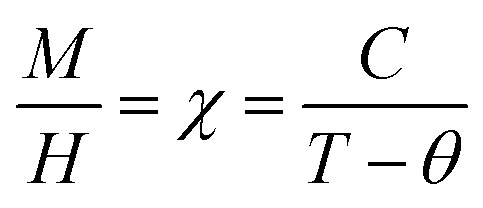
where the paramagnetic Curie temperature is symbolized by *θ* and the Curie constant is denoted as *C*. However, there was a notable disparity between the experimentally observed and theoretically fitted curves of the ZCO thin films (as illustrated in Fig. 9(a)). This discrepancy indicates that the susceptibilities *χ vs. T* did not conform to the Curie–Weiss equation. Consequently, it could be concluded that the ZCO thin films did not exhibit paramagnetic characteristics within the temperature range of 5 to 300 K. Furthermore, we could not fit the *M vs. T* data of the FC curve of the ZCO thin films only using a three-dimensional (3D) spin-wave model. However, we managed to achieve a successful fit of the *M vs. T* data obtained in the FC state within the temperature range of 5–300 K by employing a combination of the Curie–Weiss and spin-wave models. This was accomplished by applying eqn (4):^62^4
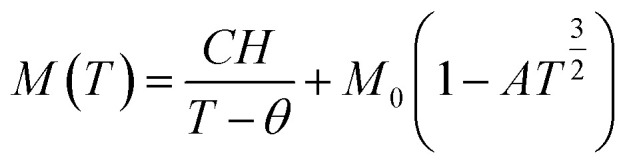
where *A* is a coefficient associated to the material structural characteristics, *C* is the Curie constant and is the paramagnetic Curie temperature, and *M*_0_ is the saturation magnetization at *T* = 0 K caused by the ferromagnetic component. According to Fig. 9(a), the 3D spin wave, and Curie–Weiss models, *CH* = 4 × 10^−4^ emu K cm^−3^ Oe^−1^, *M*_0_ = 2 × 10^−5^ emu cm^−3^, and *A* = 3 × 10^−5^ K^−3/2^, which adequately fitted the *M vs. T* curve. The PM and ferromagnetic (FM) phases coexisted concurrently in the ZCO NPs, as shown by the well-fitting curve. These magnetic observations were well supported by the bound magnetic polaron (BMP) model. According to the sample *M vs. H* loops, which were recorded at 10 and 300 K and are depicted in Fig. 10(a), non-linearity in magnetization existed up to a maximum field of 10 000 Oe, and as a result, hysteresis behavior was observed at these temperatures. The hysteresis loops shown in the inset in Fig. 10(a) yielded the highest magnetization remanence of 1.4 × 10^−4^ emu cm^−3^, coercive field value of 75 Oe for 300 K, and 1 × 10^−3^ emu cm^−3^, and coercive field value of 43 Oe for the 10 K case. The measured coercivity value indicated the presence of FM ordering at RT, but the primary contributor was paramagnetic (PM) interactions, as shown by the almost linear behavior of the *M vs. H* loop above the applied magnetic field, 2000 Oe at 10 K, while the primary contributor was diamagnetic (DM) interactions at 300 K. We fitted the measured beginning data of *M* against *H* curves recorded at 10 K and 300 K in terms of the bound magnetic polaron (BMP) model to understand the applicability of the BMP model for explaining the observed FM contribution.^63^

**Fig. 11:**
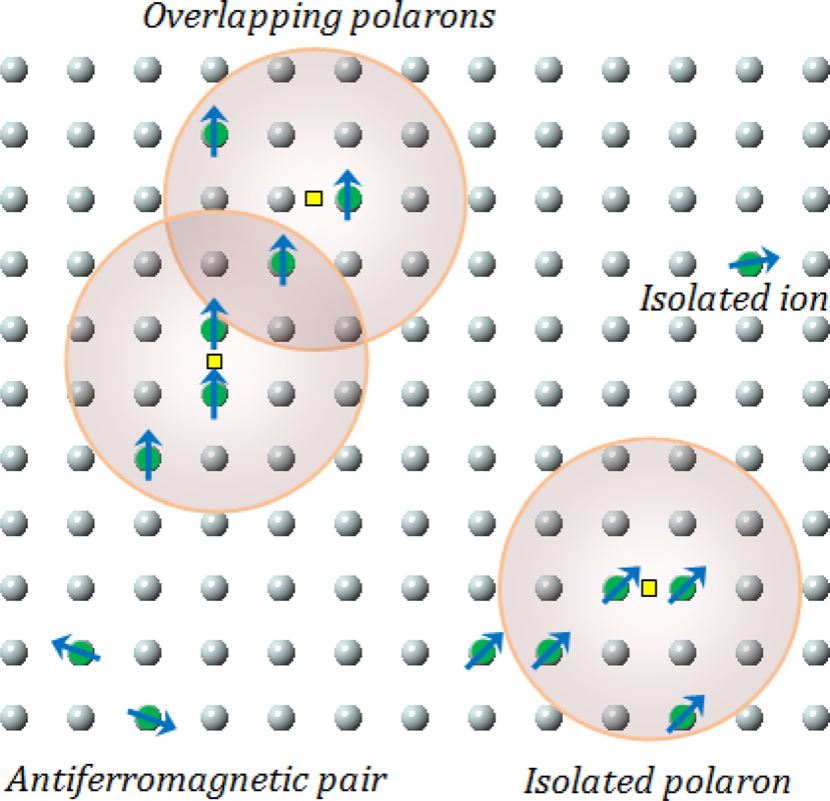
Representation of bound magnetic polarons. Cation sites are shown by small circles. Oxygen is not shown, while the unoccupied oxygen sites are represented by squares.

According to the BMP model, the presence of both correlated (as a result of BMP overlap) and isolated spins reveals how the system got magnetized. The spin-localized charge carriers powerfully interacted with the doped R-ions in the case of a correlated system. Paramagnetism (isolated spin) was caused by the portion of doped Co^2+^ ions that were not involved in the BMP interaction (schematically shown in Fig. 11). The measured magnetization may be fitted to the relation^64^ using this method, as per the following equation.5
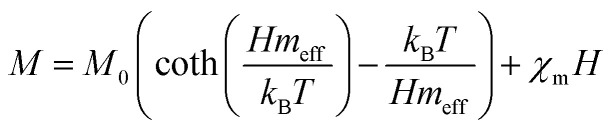


**Fig. 12:**
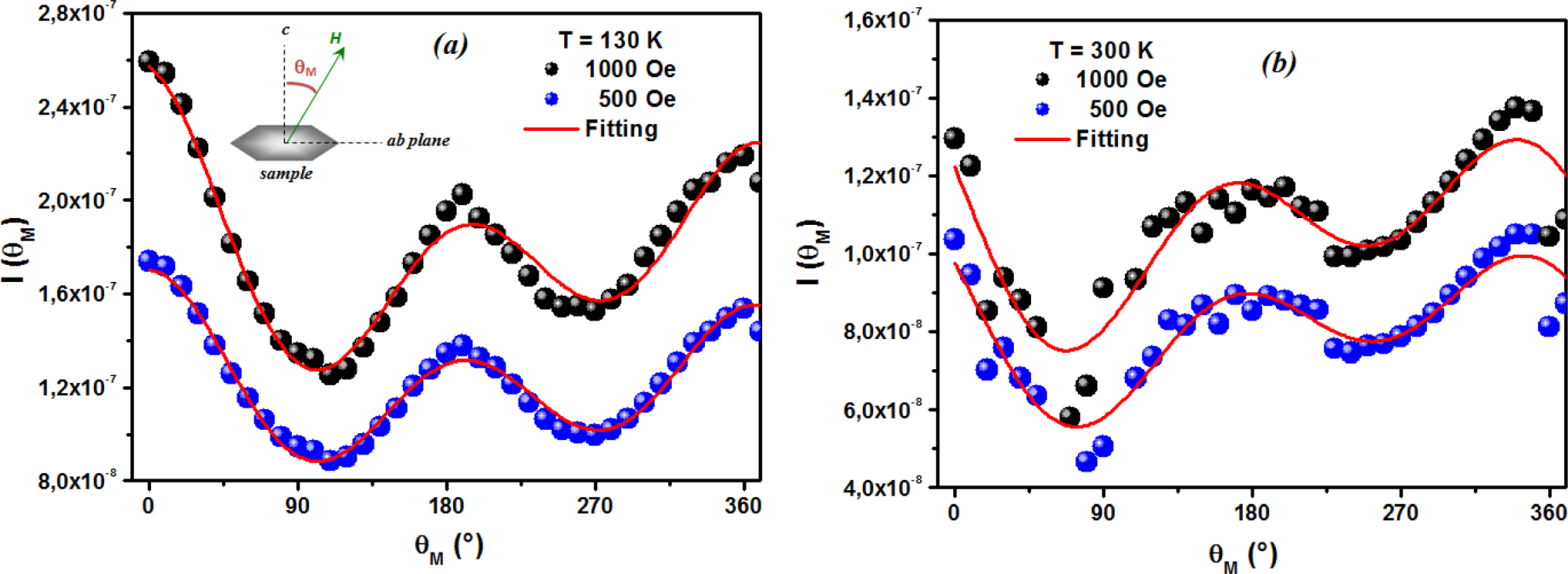
Angular dependence of the magnetic torque measured in 500 Oe and 1000 Oe magnetic fields. The classical plot (a) and polar plot (b) show selected curves at specific magnetic regimes at 130 K and 300 K.

The first term in this case represents the contribution from the BMP, while the PM and/or DM matrix is responsible for the second term. Here, *M*_0_ = *Nm*_s_, where *N* is the number-per-unit-volume and *m*_s_ is the effective spontaneous moment per BMP. Also, *m*_eff_ is the effective spontaneous magnetic moment of each BMP. As a result, the fitting operation was carried out under the assumption that *m*_s_ = *m*_eff_.^65,66^ In this case, we overlooked the interaction between the BMPs. The number of connected spins rose as a result of the BMP overlap, greatly increasing the magnetism. Previous studies have shown that the TM site in Co-doped ZnO does not produce FM.^67^ The authors confirmed the presence of itinerant electrons in defects, such as oxygen vacancies. Co caused delocalized magnetic moments, which improved the magnetic characteristics. Theoretical investigations showed that Co can contribute significantly to ferromagnetism by causing a noticeable alteration of the band structure of host oxides.^68,69^ The exchange interaction resulting among O vacancies and the dopant ions assembles a number of dopant spins around the oxygen vacancies, resulting in the formation of BMPs. As a result, the density of oxygen vacancies is proportional to the number of BMPs.^70^ The measured first *M vs. H* curves and fitted ZCO data for the BMP model are shown in Fig. 10(b). The BMP model fitted each set of experimental data rather well, and the parameters *M*_0_, *m*_eff_, *χ*_*m*_, and *N* were generated from the model's *M vs. H* curve fitting and are listed in Table 2. The paramagnetic susceptibility (*χ*_*m*_), as determined by the BMP fitting, was in the order of 10^−7^ in cgs unit, and its value varied slightly with temperature. At 10 K, it was discovered that the effective spontaneous instant per BMP, or *m*_eff_, was of the order of 8.914 10^−24^ Am^2^, rising to 9.15 10^−21^ Am^2^ at 300 K. The strength of the polaron–polaron interactions increased with the temperature, which caused a significant fall in the number of BMPs per unit volume. At 10 K, the total number of BMPs was determined to be in the order of 10^26^ cm^−3^. However, this concentration was quite high in comparison to the concentration threshold of 10^20^ cm^−3^ required for long-range percolation,^68^ which consolidates the idea of the existence of FM interactions among other different components. The presence of Co^2+^ in ZnO matrix may lead to an increase in V_O_. However, the overall numbers of BMPs responsible for long-range FM ordering was still insufficient, suggesting there were several contributors. The V_O_ defect-mediated BMPs and their percolations are important parameters that govern FM behavior. This paper extends the utility of the BMP model and provides an overview of the process governing dilute ferromagnetism. Within our study, we performed a series of angular-dependent magnetization scans within the orthogonal plane, perpendicular to the *ab* plane (Fig. 12(a)). Theses scans were carried out at *T* = 130 K and 300 K under 500 Oe and 1000 Oe magnetic fields. The angular-dependent magnetic torque measurements in identical magnetic fields of 500 Oe and1000 Oe at various temperatures are shown in Fig. 12(b). Angular-dependent magnetic torque provides information mainly about the magneto-crystalline anisotropy strength. In contrast, the angular-dependent magnetization measurements show the size of the magnetic moment at the given orientation of the sample with respect to the direction of the external magnetic field. In respect to the hexagonal symmetry, we fitted the magnetic torque, using the following formula:^71^

**Fig. 13:**
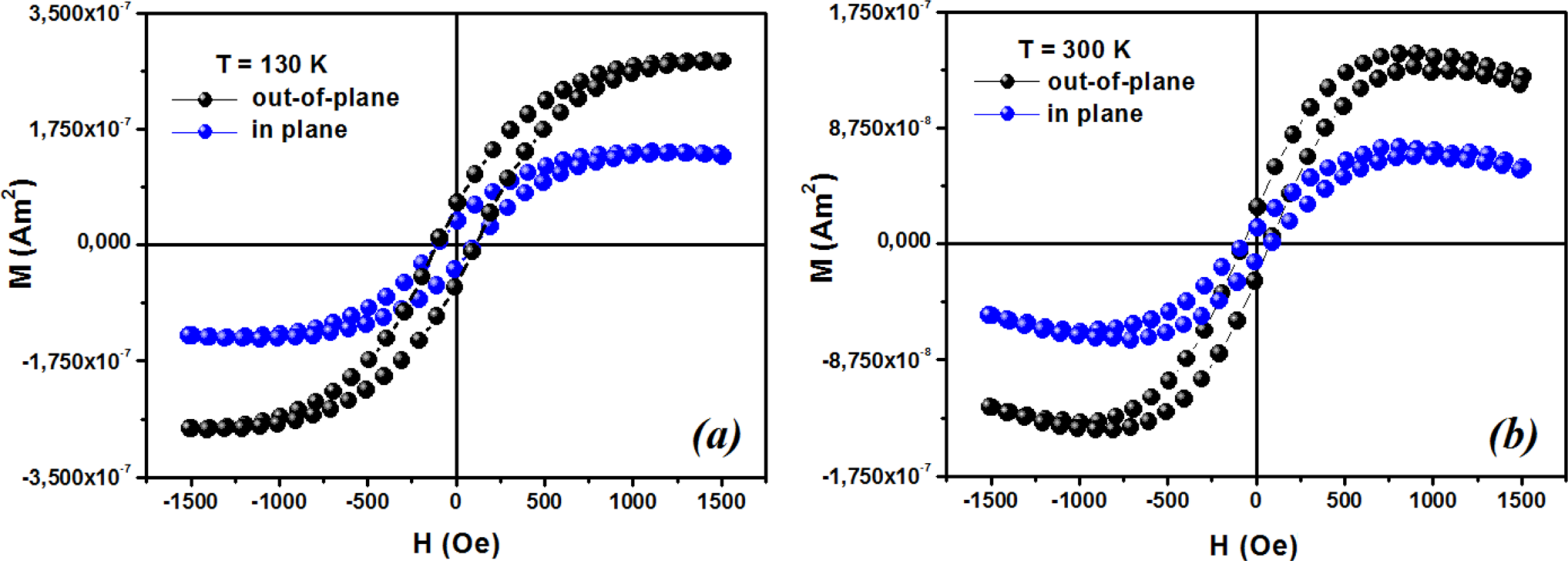
Typical magnetic hysteresis loops of ZCO thin films in the range of 0° (out-of-plane) and 90° (in-plane) at (a) 130 K and (b) 300 K.

6 *I*(*θ*_*M*_) = *T*_0_ + *T*_1_ sin[2(*θ*_*M*_ − *m*_2_)] + *T*_2_ sin[4(*θ*_*M*_ − *m*_4_)]

where *T*_0_ comes from the background of the torque magnetometer, and *T*_1,2_ are different in magnitude and likely induced the interplay of the (two and four-fold) anisotropy ratio. The coefficients of the two-fold symmetry component sin[2(*θ*_*M*_)] are terms for the UMA. The two-fold symmetry of the torque curve indicates a two-fold symmetry of the axis of magnetization (easy and hard). The four-fold symmetry component sin[4(*θ*_*M*_)] is a term for a hexagonal system. The estimated values of *T*_0_ for *T* = 130 K were 1.744(1) 10^−7^ Am^2^ and 1.185(1) 10^−7^ Am^2^ for the 500 Oe and 1000 Oe magnetic fields, respectively. For 300 K, they were 1.059(1) 10^−7^ Am^2^ and 0.795(1) 10^−7^ Am^2^ for the 500 Oe and 1000 Oe magnetic fields, respectively. Previous reports^72,73^ provided a description of the magnetization anisotropy. We carried out analysis of the out-of-plane and in-plane hysteresis loops for ZCO thin films at 130 K and 300 K (Fig. 13). Many theories exist to explain the origin of this anisotropy in magnetization. One possible explanation is the influence of magneto-crystalline anisotropy on the atomic magnetic moments. In the direction of easy magnetization, the anisotropy works toward maintaining the alignment of the individual spins, resulting in a narrow cone along the easy axis. Consequently, the magnitude of magnetization increases. Conversely, in the hard direction, the anisotropy amplifies the angular deviation of spins, causing a wider cone along the hard axis. As a result, the magnitude of magnetization decreases.

Magnetic parameters determined from the *M*–*T*, and *M*–*H* curves along with their fitting parameters obtained from the BMP model for ZCO thin films at 5 and 300 K

**Table:** 

*M*–*T* parameters			
*M*(0) × 10^−7^ (Am^2^)	*A* × 10^−5^ (K^−3/2^)	*C* × 10^−6^(Am^2^ K Oe^−1^)	*θ* (K)
1.5317	3	7.296	−1

**Table:** 

*M*–*H* parameters		
*T* (K)	*H*_C_(Oe)	*M*_r_ × 10^−7^ (Am^2^)
5	190	12.570
300	190	1.0424

**Table:** 

Fitting parameters extracted from the BMP model				
*T* (K)	*M*_0_ × 10^−5^ (Am^2^)	*m*_eff_ × 10^−21^ (Am^2^)	*χ*_*m*_ × 10^−7^ (cgs)	*N* × 10^13^ (cm^−3^)
10	2.15406	0.008914	2.39389	—
300	0.04332	9.15	−0.95792	4.734

## 4. Conclusion

The modified sol–gel approach was successfully used to synthesize ZCO nanoparticles. The obtained powder was deposed on a suprasil substrate by a pulsed laser technique (PLD). According to structural investigations using XRD and Raman spectroscopy, the ZCO nanoparticles have a wurtzite structure, with the primitive unit cell being a hexagonal system with the space group *P*6_3_*mc*. Additionally, the oxygen vacancies present in ZCO were shown by the peak at 536 cm^−1^ seen in the Raman spectra. UV-vis-NIR spectroscopy was performed to confirm the nature of the ZnO NPs powder and film. The chemical states of oxygen and zinc in the ZCO thin film were also investigated by X-ray photoelectron spectroscopy (XPS). The current work proves that BMPs are produced when oxygen defects result after annealing in vacuum and Co^2+^-ion doping. The link between BMPs causes FM in ZCO thin films. The combination of 3D spin-wave method and Curie–Weiss law provided a good match for the temperature-dependent magnetization curve of ZCO and suggested that paramagnetic and ferromagnetic phases coexist in the range of 5 to 300 K. Based on a phenomenological model, we revealed that the structure gives rise to uniaxial magneto-crystalline anisotropy (UMA) with the magnetic easy axis parallel to the *c*-axis.

## Data availability

The authors confirm that the data used to support the findings of this study are included within the article and are available from the corresponding author upon reasonable request.

## Author contributions

N. Khlifi, N. Ihzaz and L. El Mir: conceptualization, literature review, performed study, data analysis and writing of original draft, Md. O. Toulemonde, A. Dandre and C. Labrugère-Sarroste: data curation, discussion, software and validation, M. N. Bessadok, O. M. Lemine: supervision, and reviewing the manuscript.

## Conflicts of interest

The authors declare no conflict of interest.
